# Citrullination Alters the Antiviral and Immunomodulatory Activities of the Human Cathelicidin LL-37 During Rhinovirus Infection

**DOI:** 10.3389/fimmu.2020.00085

**Published:** 2020-02-04

**Authors:** Víctor Casanova, Filipa Henderson Sousa, Priyanka Shakamuri, Pavel Svoboda, Chloé Buch, Mathilde D'Acremont, Maria A. Christophorou, Jan Pohl, Craig Stevens, Peter G. Barlow

**Affiliations:** ^1^School of Applied Sciences, Edinburgh Napier University, Edinburgh, United Kingdom; ^2^Biotechnology Core Facility Branch, Division of Scientific Resources, US Centers for Disease Control and Prevention, Atlanta, GA, United States; ^3^MRC Human Genetics Unit, Institute of Genetics and Molecular Medicine, Western General Hospital, University of Edinburgh, Edinburgh, United Kingdom

**Keywords:** rhinovirus, virus, inflammation, peptide, LL-37, cathelicidin, citrullination

## Abstract

Human rhinoviruses (HRV) are the most common cause of viral respiratory tract infections. While normally mild and self-limiting in healthy adults, HRV infections are associated with bronchiolitis in infants, pneumonia in immunocompromised patients, and exacerbations of asthma and COPD. The human cathelicidin LL-37 is a host defense peptide (HDP) with broad immunomodulatory and antimicrobial activities that has direct antiviral effects against HRV. However, LL-37 is known to be susceptible to the enzymatic activity of peptidyl arginine deiminases (PAD), and exposure of the peptide to these enzymes results in the conversion of positively charged arginines to neutral citrullines (citrullination). Here, we demonstrate that citrullination of LL-37 reduced its direct antiviral activity against HRV. Furthermore, while the anti-rhinovirus activity of LL-37 results in dampened epithelial cell inflammatory responses, citrullination of the peptide, and a loss in antiviral activity, ameliorates this effect. This study also demonstrates that HRV infection upregulates PAD2 protein expression, and increases levels of protein citrullination, including histone H3, in human bronchial epithelial cells. Increased *PADI* gene expression and HDP citrullination during infection may represent a novel viral evasion mechanism, likely applicable to a wide range of pathogens, and should therefore be considered in the design of therapeutic peptide derivatives.

## Introduction

Human rhinovirus (HRV) is the most common cause of upper respiratory tract infection and a causative agent of the common cold (1). HRV infections are associated with bronchiolitis in infants and children, as well as fatal pneumonia in elderly or immunocompromised individuals (1, 2). HRV infection can also result in exacerbations of pre-existing respiratory conditions, such as asthma and chronic obstructive pulmonary disease (COPD) (3, 4). In addition, children who experience wheezing as a result of HRV infection are at increased risk of subsequently developing asthma (5). Around 150 different serotypes of HRV have been identified so far and collectively, these represent a significant burden of morbidity and mortality as well as substantial healthcare costs (6). There are currently no effective therapeutics, or preventative vaccines, licensed for use to treat or prevent HRV infection.

HRV are non-enveloped, single stranded RNA (ssRNA) viruses of the *Picornaviridae* family. All serotypes of HRV use receptor-mediated endocytosis (via ICAM-1 or LDLR) to infect upper airway epithelial cells where viral replication occurs (7, 8). Following HRV infection, cells secrete inflammatory mediators such as IL-1β, IL-6, and IL-8, which correlate with the severity of symptoms and neutrophil infiltrates (9, 10). Airway epithelial cells and infiltrating leukocytes can also respond to viral infection with the secretion of host defense peptides (HDP), also known as antimicrobial peptides (11, 12).

One of the best characterized HDP with potent antiviral activity is the human cathelicidin LL-37. LL-37 is the active cleaved fragment of the sole human cathelicidin, hCAP-18 (13). This peptide is primarily found in neutrophil specific granules but can also be produced and secreted by various cell types including epithelial cells, macrophages and lymphocytes (14). Altered Cathepsin C-mediated processing of hCAP-18, or neutropenic deficiency of LL-37 in humans is reflected by increased susceptibility to infection (15, 16), and mice lacking the cathelicidin gene exhibit increased susceptibility to both bacterial and viral infections (17, 18). Conversely, exogenous delivery of LL-37 in murine models offers increased protection against, and enhanced resolution of, respiratory viral infections (18, 19).

LL-37 concentrations are increased at sites of inflammation, including the lungs, where the peptide can act directly against a range of respiratory pathogens including influenza virus (IAV), *Mycobacterium tuberculosis, Pseudomonas aeruginosa*, and Respiratory Syncytial Virus (RSV) (19–21). The size, amphipathicity and cationic charge of LL-37 are all important determinants of its direct antimicrobial activity (22) and the five arginine residues in LL-37 critically contribute to the cationic nature of the peptide. In this regard, the core region of LL-37 [20 residues, (13–32)], which includes three arginine residues, is required for its antiviral activity against both IAV and RSV and may also be critical in the antiviral activity of the peptide against other pathogens (20, 23, 24).

Recent work has shown that posttranslational modifications can alter the structure and function of a wide range of HDP (25). Citrullination or deimination is the post-translational conversion of arginine residues within a protein or peptide to the non-coded and neutrally charged amino acid citrulline. This results in the loss of 1 positive charge per arginine converted, which can substantially alter protein folding, structure, and function (26). Citrullination is mediated by a family of calcium binding enzymes, peptidylarginine deiminases, or PADs (EC 3.5.3.15). Five different isoforms of PAD enzymes exist in humans (PAD1, 2, 3, 4, and 6), with varying substrate specificity and tissue distribution (26). While structural proteins such as vimentin, filaggrin, or fibronectin are among the most studied substrates of PAD enzymatic activity, secreted and nuclear proteins such as chemokines and histones are also targets of PAD enzymes (26, 27).

Protein citrullination and PAD activity are important in a number of physiological processes, such as epidermis differentiation, apoptosis, gene transcription or Neutrophil Extracellular Trap (NET) formation (26). However, auto-antibodies against citrullinated proteins are a hallmark of rheumatoid arthritis, and abnormally elevated levels of citrullinated proteins and PAD enzymes are found in a number of inflammatory pathologies (28). It is known that PAD2 and PAD4, together with total citrullinated protein levels, are elevated in the lungs under inflammatory conditions such as tobacco smoking, COPD, or exposure to nanoparticles (29–33). Importantly, blocking PAD enzymatic activity has been shown to reduce inflammation in several mouse models of inflammation, including allergic airway inflammation (34).

PAD2 and PAD4 isoforms have been shown to citrullinate LL-37 *in vitro*, profoundly disrupting the direct antibacterial activity of LL-37 (30) and the well-established capacity of the peptide to reduce inflammation in response to lipopolysaccharide (LPS) (35). Furthermore, citrullination results in increased susceptibility of LL-37 to degradation by bacterial or host cell proteases (30).

Previous work from our group, and others, has shown that LL-37 has direct antiviral activity toward HRV viruses (36–38). Given that citrullination can profoundly affect the functions of LL-37, and that PAD activity is increased under inflammatory conditions, we aimed to establish the impact of citrullination on the antiviral activity of LL-37 against HRV. Furthermore, we investigated whether infection with HRV results in increased *PADI* gene and PAD protein expression and increased protein citrullination in human bronchial epithelial cells.

We show that citrullination of LL-37 abrogates its direct activity against HRV and that bronchial epithelial cells display increased PAD enzyme expression and protein citrullination upon HRV infection. This work describes functional changes to the antiviral and immunomodulatory activities of LL-37 as a consequence of a posttranslational modification that is relevant *in vivo* and may reveal a novel mechanism that viral pathogens employ to avoid the actions of HDP.

## Materials and Methods

### Cell Culture and Reagents

Human bronchial epithelial cells (16HBE14°−) were obtained from Professor Dieter Gruenert (UCSF). Cell culture vessels were coated with 100 μg/ml bovine serum albumin fraction V (Sigma Aldrich, Dorset, UK), 0.5 μg/ml Cultrex mouse collagen IV (R&D Systems, Abingdon, UK), and 1 μg/ml human fibronectin (EMD Millipore, Hertfordshire, UK) before seeding. Cells were grown in Iscove's Modified Dulbecco's Medium (IMDM), GlutaMAX™ supplemented with 10% FBS (fetal bovine serum) and 1% Penicillin-Streptomycin (Thermo-Fisher, Loughborough, UK). WI-38 fetal lung fibroblasts and Hela cells were obtained from ATCC. These cells were grown in Dulbecco's modified Eagle's medium (DMEM) with 10% FBS and 1% Penicillin-Streptomycin (Thermo-Fisher, Loughborough, UK). All cells were grown in a heated humidified incubator at 37°C, 5% CO_2._ Cells were detached with 0.05% Trypsin-EDTA solution (Thermo-Fisher, UK) and seeded at 5 × 10^4^ cells/ml for 12-well plates and allowed to grow for 24 h before infection or stimulation experiments. RNAzol-RT and Saponin were all obtained from Sigma-Aldrich (Dorset, UK).

### Antibodies

Antibodies against Citrullinated Histone H3 (ab5103), PAD2 (ab16478), PAD4 (ab128086), PAD3 (ab183209), Tubulin and β-actin (ACTN05) were purchased from Abcam (Cambridge, UK). The anti-peptidyl-citrulline antibody, clone F95 (MABN328) was from EMD Millipore (Hertfordshire, UK). Antibodies against total histone H3 (1B1B2) and (96C10) were from Cell Signaling Technologies (Hertfordshire, UK). PAD1 (HPA062294) was from Sigma Aldrich (Dorset, UK), and PAD2 (12110-1-AP) was from Proteintech (Manchester, UK). All secondary antibodies used were raised in goat. F(ab')2 anti-Rabbit IgG A647, F(ab')2 anti-Mouse IgG PE (A10543), anti-Mouse IgG A594 (A11032) and anti-Mouse IgG A488 (A11029) were from Thermo Fisher Scientific (UK). Anti-Mouse IgM Texas Red was from VectorLabs (Peterborough, UK) and Anti-Rabbit IgG FITC was from Sigma Aldrich (Dorset, UK).

For flow cytometry, the following mouse anti human antibodies CD3 PEcy7, CD16 PerCP-Cy 5.5, CD19 BV786, CD56 BV650, and CD14 PE were obtained from BD Biosciences (Oxford, UK).

### Isolation of Human PBMCs

Human venous blood was collected in accordance with local institutional ethical and health & safety regulations. Peripheral blood mononuclear cell (PBMC) and granulocyte cell fractions were separated from the blood of healthy donors using dextran sedimentation (Pharmacosmos, Reading, UK) followed by discontinuous isotonic Percoll gradient centrifugation (GE Healthcare, Amersham, UK). Granulocytes and PBMC were washed twice in 1x DPBS without calcium or magnesium before resuspension in RPMI 1640 (Thermo-Fisher, Loughborough, UK). Purity was established by morphological assessment and FACS analysis. PBMC were seeded in triplicate at 2 × 10^5^ cells/ well in round bottom 96-well plates (Corning, Flintshire, UK) and rested for 2 h in RPMI 1640 5% FBS before RV1B exposure for 1 h in serum free RPMI media.

### Peptides

Peptides were assembled using the Fmoc/tBu solid-phase peptide synthesis approach (39) using either model 433A (Applied Biosystems, CA, USA) or model Liberty (CEM Corporation, NC, USA) automated peptide synthesizers followed by cleavage in the trifluoroacetic acid (TFA)/phenol/thioanisole/ethanedithiol/water (10:0.75: 0.5:0.25:0.5, w/w) mixture at 25°C for 90 min followed by precipitation with cold diethyl ether. The crude peptides were purified by preparative reversed-phase high-pressure liquid chromatography (RP-HPLC). The peptide purity (>98%) was confirmed by analytical RP-HPLC, and the masses were confirmed by mass spectrometry. Following lyophilization, the purified peptides were obtained in the form of their TFA salts. Stock solutions (5 mg/mL) were prepared in ultrapure DNAse/RNAse free water (Thermo-Fisher, Loughborough, UK) and stored in aliquots at −80°C. The sequences of the peptides used in this study are shown in Table 1.

**Table 1:** Sequences of native and citrullinated LL-37 peptides.

LL-37	LLGDFF**R**KSKEKIGKEFK**R**IVQ**R**IKDFL**R**NLVP**R**TES
LL-37_1Cit_	LLGDFF**(cit)**KSKEKIGKEFKRIVQRIKDFLRNLVPRTES
LL-37_2Cit_	LLGDFF**(cit)**KSKEKIGKEFKRIVQRIKDFL**(cit)**NLVPRTES
LL-37_3Cit_	LLGDFF**(cit)**KSKEKIGKEFKRIVQRIKDFL**(cit)**NLVP**(cit)**TES
LL-37_5Cit_	LLGDFF**(cit)**KSKEKIGKEFK**(cit)**IVQ**(cit)**IKDFL**(cit)**NLVP**(cit)**TES
Scrambled LL-37	RSLEGTDRFPFVRLKNSRKLEFKDIKGIKREQFVKIL

### Virus Propagation and Infection

To generate viral stocks, HRV-16 and HRV-1B (Public Health England Virus Collection, Salisbury, UK) were propagated in Hela cells grown at 33°C/5% CO_2_. Hela cells were tested and shown to express high levels of ICAM-1 and LDLR by flow cytometry. After 5 days, HRV infected cell or non-infected cells (“Hela Lysate” control) were subjected to three freeze-thaw cycles, harvested and spun to remove cellular debris (3,000 × g for 30 min) and frozen at −80°C.

Virus stocks were concentrated and partially purified (40, 41). Briefly, infected cell lysates were concentrated by centrifugation with a 100,000-molecular weight cut-off Vivaspin-20 filter (2,000 rpm for 2 h at 4°C; GE Healthcare, Amersham, UK). Soluble factors from cell origin pass through the filter while the virus is concentrated. Concentrated virus was then layered on a 30% sucrose layer (30%, w/v, pH 7.2 in DPBS) and ultracentrifugated in Quick-Seal® Polypropylene 8 mL tubes using a SW32.1Ti swinging bucket rotor (Beckman Coulter) for 3 h at 25,000 rpm at 16°C. Cell pellets were washed, resuspended in DPBS and aliquoted at −80°C.

Virus titrations were performed in sub-confluent WI-38 cultures in 96-well plates using serial dilutions from the viral stocks. Cells were infected for 2 h in serum free DMEM, washed to remove inoculum and left for 6 days in DMEM 5% FBS at 33°C/5% CO_2_ until CPE assessment was performed and TCID_50_ calculated using the Spearmen & Kärber algorithm and Reed Muench method (47).

### RNA Isolation, qPCR, and ELISA

Total RNA was extracted from 12-well plates using RNAzol RT. RNA concentrations were determined using a NanoDrop 1000 spectrophotometer (Thermo Fisher Scientific, Loughborough, UK). RNA integrity was checked using a 2100 Bioanalyzer (Agilent, UK) with RIN values routinely >8. Total RNA (1 μg) was transcribed to cDNA with a Precision RT all-in-one mix kit (PrimerDesign Ltd, Southampton, UK). cDNA (25 ng), 250 nM specific primers (Table 2) and SYBR green mastermix (PrimerDesign Ltd, Southampton, UK) were used to perform qPCR reactions in total volume of 20 μL in a StepOnePlus instrument (Applied Biosystems). A panel of six human reference genes were evaluated using a geNorm kit (PrimerDesign Ltd, Southampton, UK) and qbase^+^ (Biogazelle, Belgium) software. The geometric means of *ACTB* and *GAPDH* genes were selected as the most stable combination for normalization in 16HBE14°− cells. The 2^−ΔΔCt^ method was used and data is represented as fold change over untreated cells.

**Table 2:** qPCR Primer sequences utilized in this study.

**Target**	**Forward**	**Reverse**	**References**
PADI1	TCTACTCGGACTGGCTCTCTG	TGCTTCTTTTTGCCTGGTGTTT	This work
PADI2	GCTTTCCTCTGTCTGGTGGT	TTTCTTTGTGCCGGGGATGG	This work
PADI3	CTGCAGAGAATCGTGCGTGT	TGATCTCCAAAGTCGCGTCAA	This work
PADI4	CCATCCTGCTGGTGAACTGT	GAAGTCCTTGGGGGTCTTCG	This work
GAPDH	AAGCTCATTTCCTGGTATGACA	TCTTACTCCTTGGAGGCCATGT	(42)
ACTB	GGACTTCGAGCAAGAGATGG	AGGAAGGAAGGCTGGAAGAG	(43)
IL-6	GGTACATCCTCGACGGCATCT	GTGCCTCTTTGCTGCTTTCAC	(44)
CCL5	CAGTCGTCTTTGTCACCCGA	CGGGTGGGGTAGGATAGTGA	This work
IL-8	ACTGAGAGTGATTGAGAGTGGAC	AACCCTCTGCACCCAGTTTTC	(45)
Il-1β	ACAGATGAAGTGCTCCTTCCA	GTCGGAGATTCGTAGCTGGAT	(46)

To determine viral RNA copies in cells, total RNA was extracted as indicated above, and a one-step RT-qPCR was performed using 5 ng of total RNA as template together with a Precision OneStepPLUS Master Mix (PrimerDesign Ltd, Southampton, UK), and specific primers and FAM-taqman probes included in the Human Rhinovirus kit (PrimerDesign Ltd, Southampton, UK). RNA viral copies were extrapolated from the Ct values generated using the standard curve.

ELISA measurements of IL-6, IL-8, and CCL-5 were performed according to manufacturer's instructions (R&D Systems, Abingdon, UK).

### Western Immunoblotting

Cells were lysed in ice-cold RIPA buffer (150 mM NaCl, 1.0% IGEPAL® CA-630, 0.5% sodium deoxycholate, 0.1% SDS, 50 mM Tris, pH 8.0) from Sigma Aldrich (Dorset, UK), supplemented with protease inhibitors (cOmplete™ Mini, EDTA-free; Roche, West Sussex, UK). Lysates were subjected to sonication (3 bursts of 10 s) and centrifuged at 12,000 x *g* for 15 min at 4°C. Proteins in the soluble fraction were quantified using a Bradford Assay (Sigma Aldrich, Dorset, UK) and heated (95°C for 5 min) in SDS-PAGE loading buffer. Equal amounts of protein were separated by electrophoresis in 4–20% TGX mini-protean gels (Biorad, Hertfordshire, UK). Samples were transferred to PVDF membranes (Millipore) using a wet blotting system and visualized with Ponceau S staining (Thermo Fisher, Loughborough, UK). Membranes were blocked in 10% skimmed milk powder (Marvell) in Tris Buffered Saline (TBS) 0.1% Tween-20 (TBS-T) for 1 h at room temperature. The following primary antibodies were diluted in 3% milk TBS-T and incubated overnight at 4°C; α-citrullinated histone H3 (1/2,500), α-PAD2 (1/1,000), α-PAD4 (1/2,000), α-total histone H3 (1/2,000), α-β-actin (1/4,000). To detect protein citrullination, the F95 antibody was used at 1/1,000 with 5% BSA in TBS-T as blocking solution. Membranes were incubated with α-Mouse IgG 800CW; α-Mouse IgM 800CW or α-rabbit IgG 680IRdye secondary antibodies (LI-COR Biosciences Odyssey® Infrared Imaging System, Cambridge, UK). Alternatively, horseradish peroxidase (HRP)–conjugated antibodies (Agilent, Cheshire, UK) and ECL Plus reagents (GE Healthcare, Amersham, UK) were used. Image Studio Lite software (Licor) was used to quantify protein bands.

### Confocal Microscopy

Cells were seeded on glass chambered cell culture slides (Corning, Flintshire, UK) at 2 × 10^4^ cells/well. After the indicated exposures, cells were washed with DPBS and fixed with 4% PFA for 20 min at room temperature (RT). Cells were permeabilized with 0.1% Triton X-100 and blocked with PBS 5% normal goat serum (blocking buffer). Primary antibodies were diluted in blocking buffer and incubated overnight at 4°C. F95 and PAD2 antibodies were used at 1/100 dilution. After washing, goat anti-rabbit or mouse IgM secondary antibodies diluted in blocking buffer (1/1,000) were incubated with samples for 1 h at RT. Final mounting was performed in Vectashield aqueous mounting medium containing DAPI (Vector Laboratories, Peterborough, UK), prior to visualization on a Zeiss LSM 880 AxioObserver Z1 confocal fluorescent microscope (wavelengths: 405, 488, 594 nm, laser power 2%).

### Flow Cytometry

16HBE14°− cells were seeded at a density of 1 × 10^5^ cells/well in 12-well plates in duplicate wells per experimental condition. After treatment, cells were washed, detached with 0.05% Trypsin-EDTA, placed in FACS tubes and fixed with 4% PFA at room temperature for 15 min. Cells were washed with PBS and permeabilised by washing twice in PBS with 0.05% saponin. After a blocking step in 5% goat serum, primary antibodies were left overnight at 4°C in PBS with 0.05% Saponin and 5% goat serum. Fluorochrome conjugated secondary antibodies were added for 1 h at RT, and after final washing, cells were acquired in a FACS Celesta instrument (BD Biosciences).

## Results

### Citrullination of LL-37 Reduces the Antiviral Activity of the Peptide Toward Human Rhinovirus

**Figure 1:**
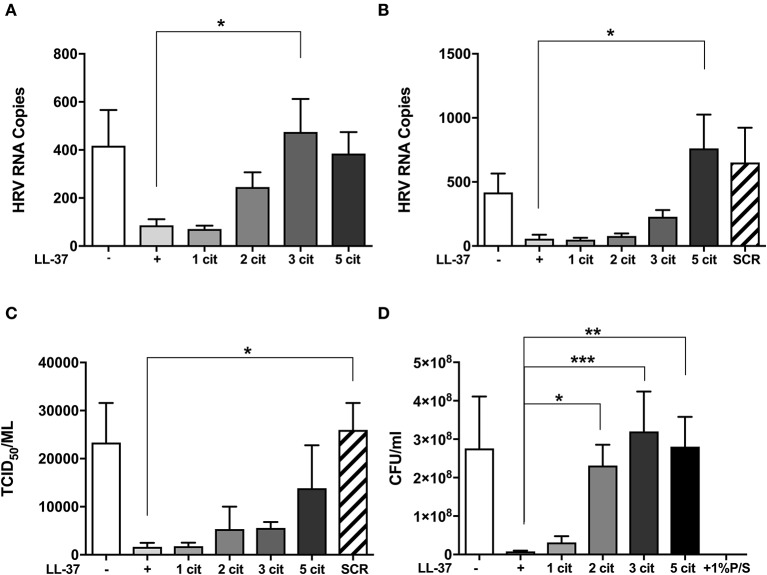
Citrullination of LL-37 abrogates the antiviral activity of the peptide toward human rhinovirus. Purified HRV1B viral particles were pre-treated with 10 μg **(A)** or 30 μg **(B)** of native or differentially citrullinated LL-37 peptides (Table 1) for 2 h before incubation with 16HBE14°− cells for 1 h at a final MOI of 1. Cells were washed to remove inoculum and incubated for 24 h before viral RNA in cells was addressed by qPCR. To measure released viral particles, cell supernatants from **(B)** were serially diluted and exposed to WI-38 cells to obtain an infectious viral titer (TCID_50_/ml) **(C)**. *S. aureus* bacteria was treated directly with 30 μg/ml of the native and modified LL-37 peptides for 2 h and incubated for 24 h before enumeration of colony formation units (CFU/ml) **(D)**. Data represent mean values ± SEM for *n* = 3 experiments for each condition *p ≤ 0.05, **p ≤ 0.01, ***p ≤ 0.001. Statistical analysis performed by one-way ANOVA with Dunnet multiple comparisons test.

Previous studies have shown that LL-37 is susceptible to citrullination by PAD enzymes, with arginines 7, 29, and 34 in the sequence being preferentially targeted (30, 35). To assess the impact of citrullination on the antiviral activity of LL-37, we generated variants of LL-37 peptides with varying numbers of arginine residues replaced with citrulline (Table 1). We then tested the ability of the modified peptides to inhibit HRV infection using the same prophylactic regime established previously (36). Briefly, RV1B virus was incubated with native or modified LL-37 peptides and after 2 h incubation the peptide/virus mixture was placed in contact with 16HBE14°− bronchial epithelial cells for 1 h to allow infection. After washing, cells were left for 24 h at 33°C to allow viral replication. Cells were harvested and HRV copy number quantified by qPCR. The addition of native LL-37 reduced HRV copy number by ~80% at both 10 and 30 μg/ml (Figures 1A,B).

Substitution of one arginine residue to citrulline (LL-37_1cit_,) did not alter the antiviral effect at either concentration tested. The substitution of two arginine residues (LL-37_2cit_) reduced LL-37 activity against HRV at 10 μg/ml (Figure 1A). However, this effect was not observed at the higher (30 μg/ml) concentration of LL-37_2cit_ (Figure 1B). Notably, LL-37_3cit_ lacked all antiviral activity at 10 μg/ml displaying a statistically significant difference to native LL-37. However, at 30 μg/ml, LL-37_3cit_, retained some activity against HRV. When all arginines in LL-37 were substituted with citrulline (LL-37_5cit_,) the antiviral effect was significantly reduced when compared to native LL-37 at 30 μg/ml, and with an apparent, but non-statistically significant loss of antiviral activity at 10 μg/ml. We also assessed a peptide with identical amino acid composition to LL-37 but with a scrambled sequence (SCR) as a control for exogenous peptide supplementation and to confirm that effects of LL-37 were sequence specific (19). The addition of LL-37-SCR at 30 μg/ml did not cause a reduction in viral copy number (Figure 1B).

To assess viral particle release from host cells, supernatants collected from the same experiments were serially diluted and used to infect WI-38 cells to measure viral titer by TCID_50_ assay. In accord with the viral copy number assessment, virus titer in cell supernatants decreased with LL-37 addition (Figure 1C) and a gradual loss of this effect was observed when two or more arginine residues were substituted with citrulline.

### Citrullination of LL-37 Abrogates the Antibacterial Activity of the Peptide Toward *S. aureus*

To address whether the impact of citrullination on LL-37 function also extended to the antibacterial activity of the peptide (30), 30 μg of native and modified peptides were incubated in direct contact with *Staphylococcus aureus (S. aureus)* for 2 h, before enumeration on an agar plate after 24 h growth. A 97% decrease in *S. aureus* CFU/ml was observed in the presence of LL-37 (Figure 1D), an antibacterial effect that was gradually reduced with each arginine to citrulline substitution, with LL-37_2cit_, LL-37_3cit_, and LL-37_5cit_ all exhibiting statistically significant differences to native LL-37 (*p* ≤ 0.05, *p* ≤ 0.01, and *p* ≤ 0.001; respectively). Taken together, these data indicate that citrullination of LL-37 profoundly affects its direct antiviral and antibacterial activity.

### Citrullination of LL-37 Alters the Immunomodulatory Activities of the Peptide

**Figure 2:**
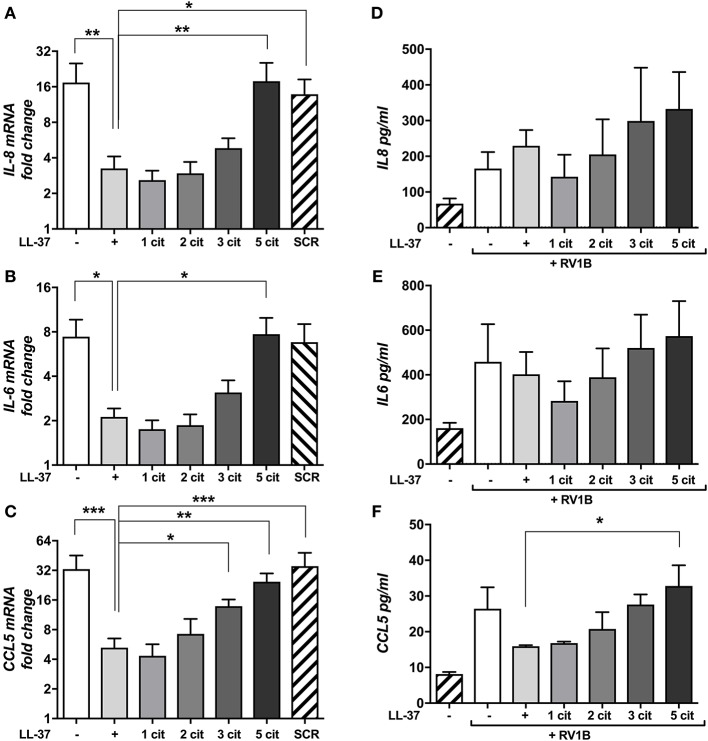
Citrullination of LL-37 modulates cytokine expression and secretion induced by human rhinovirus. Purified HRV1B viral particles were pre-treated with 30 μg **(A–C)** or 10 μg **(D–F)** of native or differentially citrullinated LL-37 peptides for 2 h and incubated with 16HBE14°− cells for 1 h at a final MOI of 1. Cells were washed to remove inoculum and left 24 h before RNA extraction. Levels of *IL-8* **(A)**, *IL-6* **(B)**, and *CCL5* **(C)** mRNA transcripts were addressed by qPCR. Values represent fold-changes over uninfected cells. Protein concentrations of IL-8 **(D)**, IL-6 **(E)**, and CCL5 **(F)** released into cell supernatants were assessed by ELISA. Data represent mean values ± SEM for *n* = 3 experiments for each condition. *p ≤ 0.05, **p ≤ 0.01, ***p ≤ 0.001. Statistical analysis performed by one-way ANOVA with Dunnet multiple comparisons test.

HRV infections are known to induce pro-inflammatory cytokine and chemokine release from epithelial cells (2) and elevated cytokines, in particular IL-8, correlate well with disease severity (9). Thus, we examined the potential for native and citrullinated forms of LL-37 to modulate HRV-induced pro-inflammatory cytokine release. We assessed mRNA expression of IL-8 (Figure 2A), IL-6 (Figure 2B), and CCL5 (Figure 2C) by qPCR. Infection with RV1B induced a marked increase in IL-8 (17-fold over untreated), IL-6 (7-fold) and CCL5 (33-fold). Addition of LL-37 reduced virus induced IL-8 mRNA expression by 81% (^*^p ≤ 0.05) and CCL5 by 84% (^***^p ≤ 0.01) and also resulted in a marked reduction of 71% in IL-6 mRNA levels (^*^p ≤ 0.05). In agreement with the effects of citrullinated LL-37 on viral copy number, LL-37_1cit_ and LL-37_2cit_ caused a reduction in the levels of IL-8, CCL5, and IL-6 mRNA induced by RV1B. Citrullination of three arginine residues in LL-37_3cit_ further reduced the immunomodulatory activity of the peptide, whereas LL-37_5cit_ completely lacked the ability to reduce IL-8, IL6, or CCL5 mRNA, showing statistically significant differences when compared to native LL-37. Addition of the LL37SCR control peptide did not result in any measurable changes in cytokine expression in response to HRV.

To assess changes at the protein level, we examined cell supernatants by ELISA (Figures 2D–F). Notably, HRV infection induced an increase in CCL5 secretion compared to untreated cells, while the addition of LL-37 reduced this effect. The effects of LL-37_1cit_ were indistinguishable from those of native LL-37, whereas LL-37_2cit_, and LL-37_3cit_ showed a moderate loss in their ability to reduce HRV-induced CCL5 secretion. LL-37_5cit_ exhibited no effect at any of the concentrations tested. However, in contrast to gene expression, the data quantifying IL-6 and IL-8 secretion was not statistically significant at this timepoint. Taken together, this data suggests that LL-37 can modulate some virus-induced proinflammatory cytokine gene expression and protein release while total citrullination of the peptide abrogates this function.

### HRV Infection or Poly I:C Stimulation Increases PAD2 Protein Levels in 16HBE14^°−^ Cells

**Figure 3:**
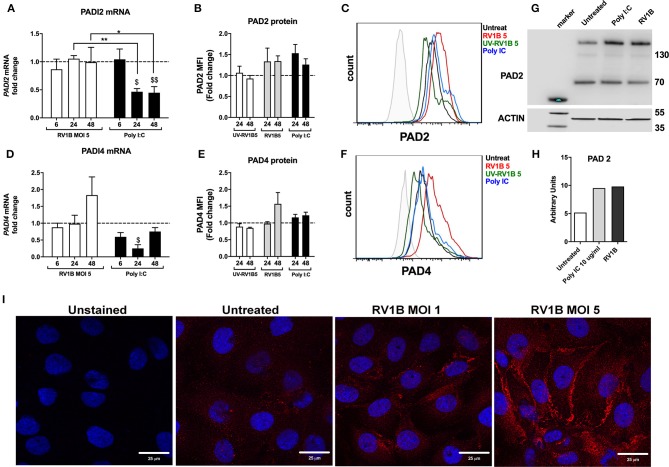
Human rhinovirus and Poly I:C stimulation increase PAD2 protein expression in lung epithelial cells. Human bronchial epithelial (16HBE14°−) cells were infected with HRV1B MOI = 5 for 6, 24 and 48 h before mRNA levels of PADI2 **(A)** and PADI4 **(D)** were assessed by qPCR. Poly I:C was used as positive control for viral dsRNA. Protein levels of PAD2 **(B)** and PAD4 **(E)** at 24 or 48 h after RV1B infection were measured by FACS (PAD2 and PAD 4) and Western immunoblotting (PAD 2 only, 24 h post infection). UV irradiated virus (UV-RV1B) was used as replication deficient virus control. Values represent fold change expression over untreated cells (dotted line) with data representing the mean ± SEM of 4 different experiments. Representative histogram plots for PAD2 **(C)** or PAD4 **(F)** protein expression in 16HBE14°− are shown. A representative Western immunoblot (representative of *n* = 3) together with quantification by densitometry are displayed **(G,H)**, showing enhanced PAD2 protein in 16HBE14°− cells after 24 h of RV1B infection at MOI = 5. Confocal microscopy images show enhanced PAD2 staining (red) in 16HBE14°− cells after 48 h of RV1B infection at MOI = 1 or MOI = 5 **(I)**. Statistical analysis in **(A,D)** was performed on ΔΔCT values via a two-way ANOVA with Tukey multiple comparisons test. ^$^*p* ≤ 0.05, ^$$^*p* ≤ 0.01 denoting significance on Poly I:C treatment vs. untreated (24 and 48 h, respectively). *p ≤ 0.05, **p ≤ 0.01.

Our results demonstrating that citrullination has a negative impact on LL-37 function together with the increased presence of PAD enzymes observed in lungs under inflammatory conditions (29–31) led to the hypothesis that HRV infection may increase levels of PAD enzymes and thus citrullination in epithelial cells. We obtained RNA and protein from 16HBE14°− cells post HRV infection and assessed PAD enzyme expression. In our model, *PADI2* mRNA was the most abundant isoform in uninfected 16HBE14°− cells, being expressed at higher levels than both *PADI1* and *PADI4*. *PADI3* mRNA levels were on the limit of detection (data not shown). Given that LL-37 is susceptible to *PADI2* and *PADI4* enzymatic activity (30), we focused upon these two isoforms. Infection of 16HBE14°− cells with HRV did not significantly alter mRNA levels of either *PADI2* (Figure 3A) or *PADI4* (Figure 3D). In contrast, protein levels of PAD2 (Figures 3B,C) and PAD4 (Figures 3E,F) appeared to moderately increase after 24 and 48 h of HRV infection, with comparable increases observed with the synthetic dsRNA analog Poly I:C as measured by FACS. Importantly, this effect was not observed when viral particles were UV-irradiated, suggesting that in the context of HRV infection, active replication of the virus is required for the increase in PAD protein observed. We used Western immunoblotting to analyse PAD2 protein levels in 16HBE14°− cells 24 h after infection and a moderate increase in PAD2 was observed after 24 h of HRV MOI 5 infection or treatment with Poly I:C (Figures 3G,H). This was confirmed through the use of confocal microscopy to analyse PAD2 protein levels in 16HBE14°− cells where a clear increase in PAD2 staining was also observed after 48 h of HRV MOI 5 infection, further confirming our observations by FACS and Western immunoblotting (Figure 3I). PAD4 protein levels, assessed by Western immunoblotting, remained unchanged (data not shown).

Interestingly, when Poly I:C was used as a synthetic analog of a dsRNA viral infection, there was a statistically significant and marked drop in *PADI2* mRNA expression at 24 h and 48 h (*p* ≤ 0.05 and *p* ≤ 0.01, respectively; Figure 3A). A similar, statistically significant drop was also observed for *PADI4* mRNA at 24 h (*p* ≤ 0.05, Figure 3D). However, protein levels of these two enzymes were moderately increased both at 24 and 48 h time points in response to Poly I:C administration at levels comparable to those elicited by HRV infection (Figures 3C,F,G,H).

### RV1B or Poly IC Stimulation Results in Increased PAD2 Expression in PBMCs

**Figure 4:**
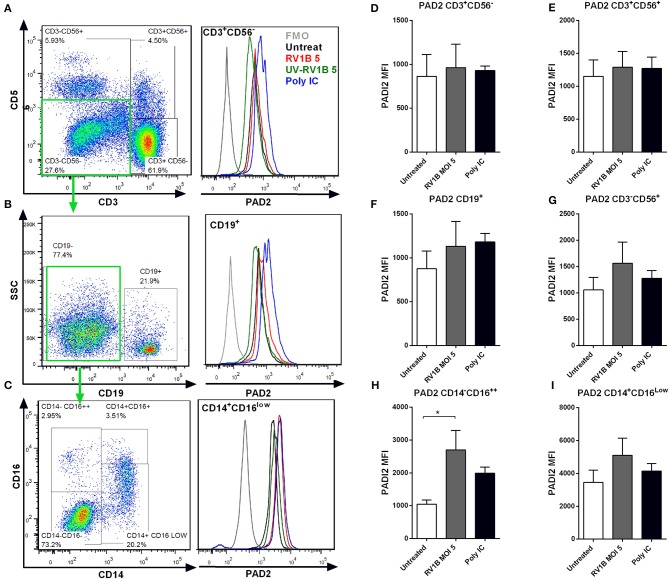
Human rhinovirus and Poly I:C stimulation increase PAD2 expression in CD14^−^CD16^++^ PBMCs. Peripheral blood mononuclear cells (PBMC) were isolated and exposed to different doses of HRV (viral MOI of 1 or 5) or to 10 μg/ml of Poly I:C for 24 or 48 h. Intracellular PAD2 expression was assessed in different PBMC subsets by FACS. Dot plots indicate the gating strategy used and histogram overlays indicate representative PAD2 levels (MFI) in different PBMCs subsets, **(A)** CD3^+^ CD56^−^ T-cells, **(B)** CD19^+^ CD3^−^ CD56^−^ B-cells, and **(C)** CD14^+^ CD16^low^ CD19^−^ CD3^−^ CD56^−^ Monocytes. Bars represent the Mean Fluorescence Index (MFI) of PAD2 levels expressed in each PBMC subset after 48 h of infection: **(D)** T-cells, **(E)** NK-T cells, **(F)** NK-cells, **(G)** B-cells, **(H)** CD14^−^ CD16^++^ monocytes, and **(I)** CD14^+^ CD16^low^ monocytes. Bars indicate the mean ± SEM of three different experiments. Statistical analysis was performed by a one way ANOVA with Tukey's multiple comparisons test (*p ≤ 0.05).

Although epithelial cells are the main site of HRV replication, immune cells are key contributors to the inflammatory response to HRV. We aimed to assess whether HRV also increased PAD2 levels in PBMCs, primary cells with well-characterized levels of PAD2 enzyme (48, 49). We obtained PBMCs from healthy donors and exposed them to RV1B or poly I:C for 24 h or 48 h. PAD2 expression was subsequently assessed in different immune subsets by FACS analysis. We found that CD14^+^ CD16^Low^ monocytes (Figure 4C) expressed higher levels of PAD2 than CD19^+^ B-cells (Figure 4B) or than CD3^+^ T-cells (Figure 4A). Interestingly, HRV (MOI 5) infection or Poly I:C stimulation resulted in a general increase in the levels of PAD2 across the different PBMC subsets analyzed. T-cells, NK-T cells and B-cells (Figures 4D-G) showed an increase in PAD2 levels at 24 h post-stimulation (data not shown), while in NK cells and monocyte subsets the increase PAD2 was more pronounced at 48 h (Figures 4F,H,I). In addition, increases in PAD2 were abolished when UV-irradiated viral particles were used as control (data not shown). A statistically significant increase in PAD2 expression was observed after 48 h RV1B MOI 5 infection, in the CD14^−^ CD16^++^ subset, confirming that HRV viral infection is also able to increase PAD2 levels in non-epithelial cells. These observations suggest that viral infection or binding of ligand to Toll-Like Receptor-3 (TLR-3) can result in altered PAD expression, which has the potential to impact on global protein citrullination.

### HRV Infection or Poly I:C Stimulation Increase Protein Citrullination in Lung Epithelium

**Figure 5:**
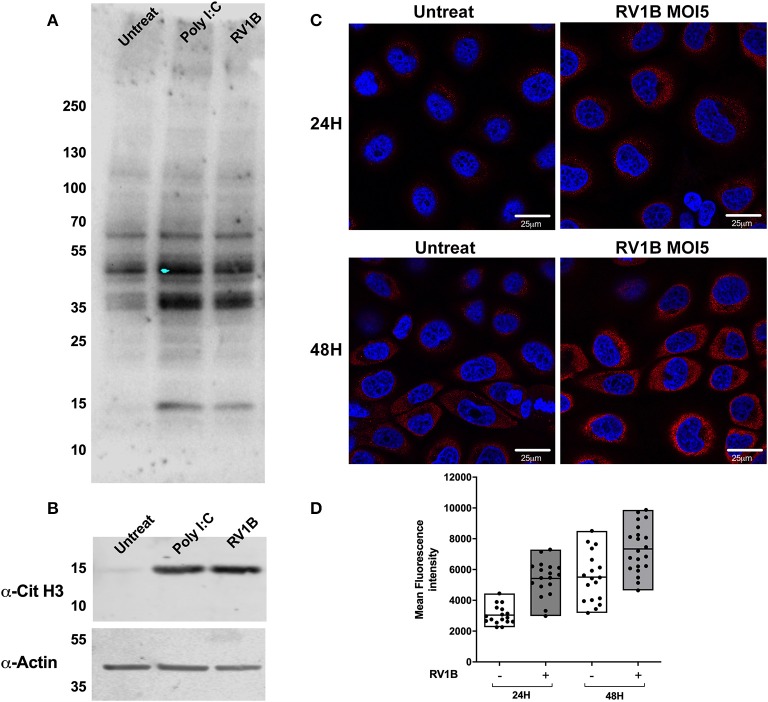
Human rhinovirus and Poly I:C stimulation increase protein citrullination in 16HBE14°− cells. Human bronchial epithelial (16HBE14°−) cells were infected with HRV1B MOI = 5 or treated with Poly IC and total citrullination was measured with a monoclonal antibody against peptidyl citrulline (F95). **(A)** After 24 h, lysates were obtained and blotted with F95 antibody, α-citrullinated histone H3, and actin, which was used as loading control. **(B)** Confocal microscopy images were also taken at 24 or 48 h after infection, showing F95 staining (RED) and DAPI as nuclear counterstaining (BLUE). **(C)** Quantification of at least four different fields of view from **(C)** is shown in **(D)**, with each dot representing a different cell and boxes displaying min to max and mean values of F95 staining intensity.

To address whether the increased levels of PAD enzymes observed after HRV infection resulted in increased protein citrullination, we used a well-characterized monoclonal antibody (F95) for the specific detection of citrullinated peptides (29, 50). Using lysates from infected, poly I:C treated and untreated cells for immunoblotting with the F95 antibody, we determined that both HRV infection and poly I:C stimulation increased the intensity of citrullinated proteins at 35–40 kDa. In addition, a clear increase in a band ~15 kDa was observed in the presence of Poly I:C and HRV treatment (Figure 5A). Because histones display a molecular weight ~18 kDa and they are well-characterized substrates for PAD enzymatic activity (51, 52), we used an antibody to detect citrullinated histone H3 (Cit H3) on the same lysates used in Figure 5A to confirm that Histone H3 was being citrullinated. A clear increase in the citH3 band intensity was observed in the presence of Poly I:C or HRV stimulation (Figure 5B), confirming that citrullination of H3 is increased under these stimuli.

We used confocal microscopy to assess total citrullinated protein levels at different time points post HRV infection (Figure 5C). After 24 and 48 h of infection, a clear increase in F95 staining and protein citrullination was detectable in the presence of HRV (Figures 5C,D). Taken collectively these data suggest that HRV infection, or activation of TLR3, results in increased protein citrullination, including histone H3.

## Discussion

Previous studies have shown PAD2 and PAD4 isoforms citrullinate LL-37 peptides *in vitro*, inhibiting the direct antibacterial activity of LL-37 (30), the ability of LL-37 to enhance nucleic acid uptake and subsequent sensing by plasmacytoid dendritic cells (53), and the ability of LL-37 to reduce inflammation in response to lipopolysaccharide (LPS) (35). Our results confirm that citrullination abrogates the antimicrobial and immunomodulatory activities of LL-37 in the context of viral infection, and further show that citrullination of LL-37 abrogates its antiviral activity, identifying a novel role for citrullination in the innate response to viral infection.

Airway epithelial cells are the main target of HRV infection and replication. In response to infection, these cells can release host defense peptides (11) which play a key role in the inflammatory and innate immune responses (12). LL-37 concentrations of ~5 μg/ml have been recovered in the bronchoalveolar lavage fluid (BAL) from uninfected infants, with levels up to ~30 μg/ml post infection (11, 54). Here we used concentrations of exogenous LL-37 that mimic physiological concentrations, and in agreement with our own recent studies (36) and others (37, 38), show that direct incubation of LL-37 with HRV prior to infection of lung epithelial cells is effective at reducing HRV RNA copy number and virion release into cell supernatants.

Inflammatory conditions that favor LL-37 release are known to increase PAD enzyme levels and protein citrullination in neutrophils (55), as well as in the lungs (29–31). PAD enzymes, in particular PAD2, are released into extracellular spaces such as cell-free BAL fluid (32) where they would encounter LL-37, therefore LL-37 citrullination is likely to occur in extracellular spaces under inflammatory conditions.

We assessed the impact of citrullination on the ability of LL-37 to reduce HRV replication and found a striking loss of antiviral activity when all arginines on LL-37 (LL-37_5cit_) were substituted with citrulline. Importantly, substituting only three of the residues LL-37_3cit_ (Arg 7, 29 and 37) also drastically reduced LL-37 antiviral activity. In contrast, substitution of only 1 residue (Arg 7) did not alter antiviral activity. While different combinations of citrullinated residues in LL-37 peptide sequence are possible, we used LL-37 peptides that are reported to be generated after *in vitro* exposure to purified PAD2 or PAD4 enzymes, with mass spectrometry data indicating that arginine residues 7, 29 and 34 are preferentially targeted by human PAD2 and PAD4 (30, 35). Each arginine conversion to citrulline reduces the net charge of LL-37 peptide, resulting in LL-37_5cit_ with a +1 net charge, as opposed to the +6 net charge of native LL-37 (30). This would affect the ability of LL-37 to interact electrostatically with negatively charged molecules such as LPS (56). Interestingly, while citrullination of LL-37 substantially reduced the antibacterial activity of the peptide against *S. aureus*, prior studies have determined that proteinases released by this pathogen have the capacity to cleave LL-37, potentially at the Arg19-Ile20 and Arg23-Ile24 bonds (57). Thus, it would be interesting to determine the susceptibility of citrullinated forms of the peptide in this context.

In line with this, LL-37_5cit_ is unable to reduce LPS-mediated release of TNF-α due to a lack of LPS-binding capacity (35). Our own data (36) suggests that the direct interaction of LL-37 with viral particles is a key requirement to reduce HRV infection, as treating viral particles with peptide prior to infection is the most effective way to reduce HRV copy numbers. Similar results have been observed with other viruses including RSV and influenza (19, 58). We believe that citrullination alters the ability of LL-37 to interact with viral particles, in doing so disrupting the antiviral activity of the peptide.

In addition to its direct antiviral activity, LL-37 has potent and broad ranging immunomodulatory activity (59). Our results show that incubation of HRV with LL-37 alters the virus-mediated gene expression of IL-8, IL-6, and CCL5 mRNA in response to infection. A reduction in viable HRV particles may explain the lower gene expression levels of proinflammatory cytokines observed, however, analysis of protein released into the supernatant determined that incubation with LL-37 did not directly correlate with significant changes at the protein level. Interestingly, pre-incubation of RSV with LL-37 was noted to limit the expression of IL-6, CCL5, and IP-10 in response to infection (58). Recent studies have also demonstrated that citrullination inhibits LL-37-mediated DNA uptake and recognition by plasmacytoid dendritic cells (53). Given that LL-37 has been shown to facilitate recognition of TLR3 ligands and enhance responses to viral RNA (60), the implications of LL-37 citrullination on enhancement of viral recognition should be a key area of focus. Our results indicate that citrullination of LL-37 may alter inflammatory and innate immune responses in infection, however further work is clearly required to fully delineate whether this is skewed toward a pro- or anti-inflammatory state in a clinical context.

We have shown for the first time that HRV infection increases PAD2 protein expression in lung epithelial cells. The increase in PAD2 protein expression is not observed when replication-deficient (UV treated) HRV is used; suggesting that PAMPs associated with viral replication may induce PAD expression. One of these PAMPs would be dsRNA intermediates that signal through TLR3. We show that a synthetic dsRNA and TLR3 agonist, poly I:C, is also capable of increasing PAD2 protein levels. Interestingly, Poly I:C stimulation decreases expression of *PADI2* and *PADI4* mRNA. This inconsistency between PAD protein and mRNA levels has been reported previously (61). An increase in PAD2 protein levels was also observed in PBMC exposed to HRV or to Poly I:C, suggesting that increases in PAD enzymes are part of immune cell activation (49, 62). This work further characterizes PAD enzyme activity in PBMC, describing for the first time, responses to a viral pathogen. The finding that monocytes are a subset with highest PAD2 levels agrees with the observation that monocytes demonstrate the strongest citrullination responses within the isolated PBMC population (63). In addition, we found statistically significant increases in PAD2 in PBMCs with a phenotype compatible with “non-classical” or “inflammatory” monocytes, a subset more prone to the release of pro-inflammatory cytokines (64), strengthening the notion that HRV-induced inflammation would increase PAD and protein citrullination. Thus, by increasing PAD2-mediated citrullination, HRV, and potentially other viruses, may be able to citrullinate LL-37, reducing its efficacy and supporting viral replication and persistence within host cells.

The total levels of citrullinated proteins in cells were also found to be increased upon HRV infection. Immunostaining patterns and multiple bands observed by immunoblotting highlight multiple proteins being citrullinated. The presence of citrullinated proteins in unstimulated cells suggests that a basal rate of protein citrullination exists, however, this is substantially increased upon HRV infection. To further confirm these observations, we made use of citrullinated histone H3 (CitH3), a well-established readout of PAD activity (42, 43, 55). We show that HRV infection or treatment of cells with poly I:C results in increased CitH3 levels. Citrullination of histone H3 has also been used as an indicator of Neutrophil Extracellular Trap (NET) release and may be of use as biomarker for certain conditions such as sepsis (44). In this regard, adapted mouse models of rhinovirus infection have shown the presence of NETS after RV1B infection, suggesting that RV1B infection results in H3 citrullination (45). Taken together, these findings suggest that viral infection does result in increased protein citrullination, both in human bronchial epithelial cell lines and in mouse models of HRV infection.

HRV infection and replication in epithelial cells results in the secretion of a variety of cytokines and chemokines which recruit immune cells to the lungs leading to neutrophil activation. It has been established that increased PAD2 protein expression and citrullinated proteins are observed in the lungs under certain inflammatory conditions, such as in response to smoking (29, 31), nanoparticles or pollutants (33) or in COPD, with PAD2 histological staining showing strong reactivity in bronchial epithelial cells and in infiltrated leukocytes (30). Our data now suggests that HRV infections would also result in increases in PAD and citrullinated protein levels in the lungs.

We propose that abnormally elevated PAD activity would result in LL-37 citrullination, thus rendering the peptide ineffective against HRV or other respiratory pathogens. Furthermore, given the immunomodulatory effects of LL-37, citrullination of the peptide could also lead to exaggerated pro-inflammatory responses (35). Together, this may contribute to secondary bacterial infections and exacerbations of pre-existing conditions observed in susceptible individuals, which are important factors in HRV epidemiology (2). In instances of viral infection, the degree to which LL-37 is citrullinated, and the specific amino acid residues that are preferentially targeted will likely reflect which PAD enzymes are induced as a result of the infection. A full assessment of PAD expression in clinical instances of viral infection may have utility for predicting altered innate responses. Therefore, we suggest that further work is required to develop better tools to characterize citrullination, particularly HDP citrullination, and to establish prevalence in other viral infections.

We anticipate the findings of this work will also apply to other host defense molecules where cationicity and arginine residues are important for antimicrobial function. In this regard, post-translational modifications affecting arginine have been described in human defensin-1 (HNP-1) (46) affecting its antibacterial activity. We and others have shown that cathelicidins show promise as antivirals against HRV, influenza, RSV and other respiratory pathogens (19, 20, 36). However, host mediated modifications of this peptide, such as citrullination, may limit its effectiveness. Significantly, another posttranslational modification affecting the cationicy of LL-37 has also been reported. Carbamylation results in the non-enzymatic conversion of lysine residues to neutral homocitrulline (65, 66) and thus reduces the net positive charge of LL-37. Similarly to citrullinated LL-37, carbamylated LL-37 also displays reduced direct antibacterial activity and reduced ability to modulate LPS-mediated cytokine release (65), confirming the importance of positive charge in the normal function of LL-37.

In summary, our data suggests that strategies oriented toward engineering citrullination resistant host defense peptides or reducing abnormally elevated levels of PAD enzyme activity may reduce the severity of HRV and other viral pathogens, and in turn reduce the risk of secondary bacterial infections and exacerbations of pre-existing pulmonary conditions.

## Data Availability Statement

The datasets generated for this study are available on request to the corresponding author.

## Ethics Statement

This study was carried out in accordance with the requirements of the Edinburgh Napier University School of Applied Sciences Research Integrity Committee. All subjects gave written informed consent in accordance with the Declaration of Helsinki.

## Author Contributions

VC conceived the study, contributed to the experimental design, performed the experimental work, and contributed to the drafting of the manuscript. FS and PSv contributed to the experimental design, performed the experimental work and contributed to the drafting of the manuscript. PSh, CB, and MD'A performed the experimental work. MC contributed to the experimental design, and contributed to the drafting of the manuscript. JP and CS contributed to the experimental design, and the drafting of the manuscript. PB conceived the study, contributed to the experimental design, and contributed to the drafting of the manuscript.

### Conflict of Interest

The authors declare that the research was conducted in the absence of any commercial or financial relationships that could be construed as a potential conflict of interest.
